# Muscle injuries and strategies for improving their repair

**DOI:** 10.1186/s40634-016-0051-7

**Published:** 2016-07-22

**Authors:** Thomas Laumonier, Jacques Menetrey

**Affiliations:** Department of Orthopaedic Surgery, Geneva University Hospitals & Faculty of Medicine, 4, Rue Gabrielle Perret-Gentil, 1211 Geneva 14, Switzerland

**Keywords:** Skeletal muscle, Injury, Regeneration, Stem cell, Fibrosis, Scaffolds, Growth factors

## Abstract

Satellite cells are tissue resident muscle stem cells required for postnatal skeletal muscle growth and repair through replacement of damaged myofibers. Muscle regeneration is coordinated through different mechanisms, which imply cell-cell and cell-matrix interactions as well as extracellular secreted factors. Cellular dynamics during muscle regeneration are highly complex. Immune, fibrotic, vascular and myogenic cells appear with distinct temporal and spatial kinetics after muscle injury. Three main phases have been identified in the process of muscle regeneration; a destruction phase with the initial inflammatory response, a regeneration phase with activation and proliferation of satellite cells and a remodeling phase with maturation of the regenerated myofibers. Whereas relatively minor muscle injuries, such as strains, heal spontaneously, severe muscle injuries form fibrotic tissue that impairs muscle function and lead to muscle contracture and chronic pain. Current therapeutic approaches have limited effectiveness and optimal strategies for such lesions are not known yet. Various strategies, including growth factors injections, transplantation of muscle stem cells in combination or not with biological scaffolds, anti-fibrotic therapies and mechanical stimulation, may become therapeutic alternatives to improve functional muscle recovery.

## Introduction

Human skeletal muscle is about 40 % of the body mass and is formed by bundle of contractile multinucleated muscle fibers, resulting from the fusion of myoblasts. Satellite cells (SC) are skeletal muscle stem cell located between the plasma membrane of myofibers and the basal lamina. Their regenerative capabilities are essential to repair skeletal muscle after injury (Hurme and Kalimo 1992; Lipton and Schultz 1979) (Sambasivan et al. 2011; Dumont et al. 2015a). In adult muscles, SC are found in a quiescent state and represent, depending on species, age, muscle location, and muscle type, around 5 to 10 % of skeletal muscle cells (Rocheteau et al. 2015). After injury, SC become activated, proliferate and give rise to myogenic precursor cells, known as myoblasts. After entering the differentiation process, myoblasts form new myotubes or fuse with damaged myofibers, ultimately mature in functional myofibers.

Skeletal muscle injuries can stem from a variety of events, including direct trauma such as muscle lacerations and contusions, indirect insults such as strains and also from degenerative diseases such as muscular dystrophies (Huard et al. 2002; Kasemkijwattana et al. 2000; Kasemkijwattana et al. 1998; Menetrey et al. 2000; Menetrey et al. 1999; Crisco et al. 1994; Garrett et al. 1984; Lehto and Jarvinen 1991; Jarvinen et al. 2005; Cossu and Sampaolesi 2007). Skeletal muscle can regenerate completely and spontaneously in response to minor injuries, such as strain. In contrast, after severe injuries, muscle healing is incomplete, often resulting in the formation of fibrotic tissue that impairs muscle function. Although researchers have extensively investigated various approaches to improve muscle healing, there is still no gold standard treatment.

This concise review provides a sight about the various phases of muscle repair and regeneration, namely degeneration, inflammation, regeneration, remodeling and maturation. We also give an overview of research efforts that have focused on the use of stem cell therapy, growth factors and/or biological scaffolds to improve muscle regeneration and repair. We also address the therapeutic potential of mechanical stimulation and of anti-fibrotic therapy to enhance muscle regeneration and repair.

## Review

### Muscle healing process

**Fig. 1:**
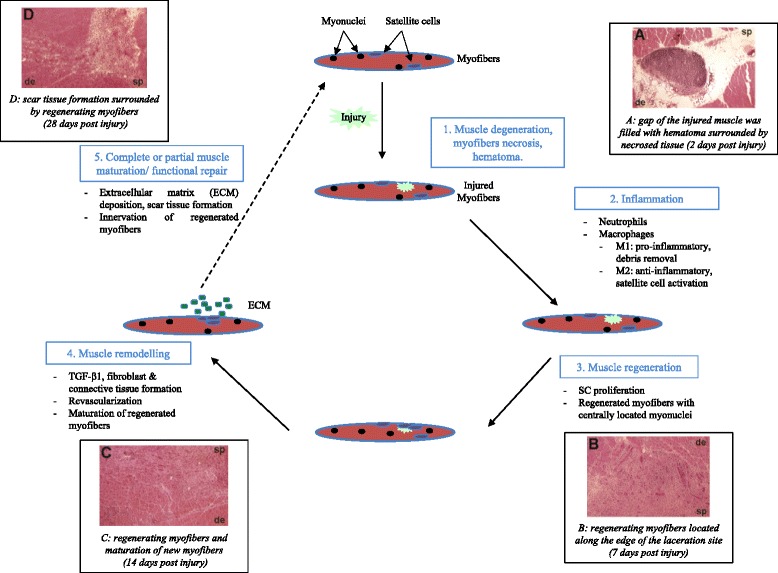
Sequential cycle of muscle healing phases after laceration. Histological images adapted from Menetrey et al, Am J Sports Med 1999. (sp: superficial portion, de: deepest part)

Skeletal muscle has a robust innate capability for repair after injury through the presence of adult muscle stem cells known as satellite cells (SC). The disruption of muscle tissue homeostasis, caused by injury, generates sequential involvement of various players around three main phases (Fig. 1).

- (1, 2) Degeneration/inflammation phase: characterized by rupture and necrosis of the myofibers, formation of a hematoma and an important inflammatory reaction.
- (3) Regeneration phase: phagocytosis of damaged tissue, followed by myofibers regeneration, leading to satellite cell activation.
- (4, 5) Remodeling phase: maturation of regenerated myofibers with recovery of muscle functional capacity (4) and also fibrosis and scar tissue formation (5).

#### Muscle degeneration and inflammation

Active muscle degeneration and inflammation occur within the first few days after injury. The initial event is necrosis of the muscle fibers, which is triggered by disruption of local homeostasis and particularly by unregulated influx of calcium through sarcolemma lesions (Tidball 2011). Excess in cytoplasmic calcium causes proteases and hydrolases activation that contribute to muscle damage and also causes activation of enzymes that drive the production of mitogenic substances for muscle and immune cells (Tidball 2005). After muscle degeneration, neutrophils are the first inflammatory cells infiltrating the lesion. A large number of pro-inflammatory molecules such as cytokines (TNF-α, IL-6), chemokine (CCL17, CCL2) and growth factors (FGF, HGF, IGF-I, VEGF; TGF-β1) are secreted by neutrophils in order to create a chemoattractive microenvironment for other inflammatory cells such as monocytes and macrophages (Tidball 1995; Toumi and Best 2003). Two types of macrophages are identified during muscle regeneration (McLennan 1996), which appear sequentially during muscle repair (Arnold et al. 2007). M1 macrophages, defined as pro-inflammatory macrophages, act during the first few days after injury,. contribute to cell lysis, removal of cellular debris and stimulate myoblast proliferation. Conversely, M2 macrophages, defined as anti-inflammatory macrophages, act 2 to 4 days after injury, attenuate the inflammatory response and favor muscle repair by promoting myotubes formation (Tidball and Wehling-Henricks 2007; Chazaud 2014; Chazaud et al. 2003). Macrophages, infiltrating injured muscle, are key players of the healing process (Zhao et al. 2016), able to participate in the muscle regeneration process or to favor fibrosis (Munoz-Canoves and Serrano 2015; Lemos et al. 2015).

### Muscle regeneration, remodeling and maturation

Muscle regeneration usually starts during the first 4–5 days after injury, peaks at 2 weeks, and then gradually diminishes 3 to 4 weeks after injury. It’s a multiple steps process including activation/proliferation of SC, repair and maturation of damaged muscle fibers and connective tissue formation. A fine balance between these mechanisms is essential for a full recovery of the contractile muscle function.

Muscle fibers are post-mitotic cells, which do not have the capacity to divide. Following an injury, damaged muscle fibers can’t be repaired without the presence of adult muscle stem cells, the satellite cells (SC) (Relaix and Zammit 2012; Sambasivan et al. 2011). Following activation, SC proliferate and generate a population of myoblasts that can either differentiate to repair damaged fibers or, for a small proportion, self-renew to maintain the SC pool for possible future demands of muscle regeneration (Collins 2006; Dhawan and Rando 2005). SC cycle progression and cell fate determination are control by complex regulatory mechanisms in which, intrinsic and extrinsic factors are involved (Dumont et al. 2015a; Dumont et al. 2015b).

#### Connective tissue/fibrosis

Connective tissue remodeling is an important step of the regenerative muscle process. Rapidly after muscle injury, a gap is formed between damaged muscle fibers and filled with a hematoma. Muscle injuries can be clinically classified depending of the nature of the hematoma (size, location). Late elimination of the hematoma is known to delay skeletal muscle regeneration, to improve fibrosis and to reduce biomechanical properties of the healing muscle (Beiner et al. 1999). In rare complication, major muscle injuries may lead to the development of myositis ossificans that will impair muscle regeneration and repair (Beiner and Jokl 2002) (Walczak et al. 2015).

The presence of fibrin and fibronectin at the injury site, initiate the formation of an extracellular matrix that is rapidly invaded by fibroblasts (Darby et al. 2016; Desmouliere and Gabbiani 1995). Fibrogenic cytokines such as transforming growth factor β1 (TGF-β1) participate to excessive fibroblasts/myofibroblasts proliferation and to an increase in type I/III collagens, laminin and fibronectin production (Lehto et al. 1985). In its initial phase, the fibrotic response is beneficial, stabilizing the tissue and acting as a scaffold for myofibers regeneration. Nevertheless, an excessive collagen synthesis post injury, often result in an increase of scar tissue size over time that can prevent normal muscle function (Mann et al. 2011). Many growth factors are involved in the development of fibrosis, such as Connective Tissue Growth Factor (CTGF), Platelet-Derived Growth Factor (PDGF) or myostatin. TGF-β1, by stimulating fibroblasts/myofibroblasts to produce extracellular proteins such as fibronectin and type I/III collagen, has been identified as the key element in this process (Mann et al. 2011),. Although fibroblasts are the major collagen-producing cells in skeletal muscle, TGF-β1 have also an effect directly on myoblasts causing their conversion to myofibroblasts. Thus myoblasts initially acting to repair damaged myofibers, will produce significant level of collagen and will contribute to muscle fibrosis (Li and Huard 2002).

#### *Revascularization*

The restoration of the blood supply in the injured skeletal muscle is one of the first signs of muscle regeneration and is essential to its success. Without revascularization, muscle regeneration is incomplete and a significant fibrosis occurs (Best et al. 2012; Ota et al. 2011). After muscle trauma, blood vessels rupture induces tissue hypoxia at the injury site (Jarvinen et al. 2005). New capillaries formation quickly after injury is therefore necessary (Scholz et al. 2003) for a functional muscle recovery. Secretion of angiogenic factors such as vascular endothelial growth factor (VEGF) at the lesion site is important and several studies have shown that VEGF, by favoring angiogenesis, improve skeletal muscle repair (Deasy et al. 2009; Frey et al. 2012).

#### Innervation

Muscle repair is complete when injured myofibers are fully regenerated and become innervated. The synaptic contact between a motor neuron and its target muscle fiber, often take place at a specific site in the central region of myofibers, the neuromuscular junction (NMJ) (Wu et al. 2010). NMJ are essential for maturation and functional activity of regenerating muscles. Within 2–3 weeks after muscle damage, the presence of newly formed NMJ is observed in regenerative muscle (Rantanen et al. 1995; Vaittinen et al. 2001).

### Strategies to improve muscle regeneration and repair

#### Growth factors

Growth factors play a variety of roles in the different stages of muscle regeneration (Grounds 1999; Menetrey et al. 2000). These biologically active molecules, synthetized by the injured tissue or by other cell types present at the inflammatory site, are release in the extracellular space and modulate the regenerative response (Table 1). Although hepatocyte growth factor (HGF), fibroblast growth factor (FGF) and platelet-derived growth factor (PDGF) are of interest because of their capacity to stimulate satellite cells (Sheehan et al. 2000; Allen and Boxhorn 1989; Yablonka-Reuveni et al. 1990), insulin like growth factor-1 (IGF-I) appears to be of particular importance for the muscle regeneration process. IGF-I stimulates myoblasts proliferation and differentiation (Engert et al. 1996) and is implicated in the regulation of muscle growth (Schiaffino and Mammucari 2011). In a mouse model, direct injections of human recombinant IGF-I at two, five, and seven days after injury enhanced muscle healing in lacerated, contused, and strain-injured muscles (Menetrey et al. 2000; Kasemkijwattana et al. 2000). However, the efficacy of direct injection of recombinant proteins is limited by the high concentration of the factor typically required to elicit a measurable effect. This is mainly due to the bloodstream’s rapid clearance of these molecules and their relatively short biological half-lives. Gene therapy may be an effective method by which to deliver high, maintainable concentrations of growth factor to injured muscle (Barton-Davis et al. 1998; Barton et al. 2002; Musaro et al. 2001). Although IGF-I improved muscle healing, histology of the injected muscle revealed fibrosis within the lacerated site, despite high level of IGF-I production (Lee et al. 2000). Another growth factor, VEGF, by favoring angiogenesis, is known to enhance skeletal muscle repair (Deasy et al. 2009; Frey et al. 2012; Messina et al. 2007). By targeting simultaneously angiogenesis and myogenesis, it was shown that combined delivery of VEGF and IGF-I enhance muscle regenerative process (Borselli et al. 2010). In this direction, the use of platelet-rich plasma (PRP) is considered as a possible alternative approach based on the ability of autologous growth factors to improve skeletal muscle regeneration (Hamid et al. 2014; Hammond et al. 2009). Considered as safe products, autologous PRP injections are increasingly used in patients with sports-related injuries (Engebretsen et al. 2010). Nevertheless, a recent randomized clinical trial show no significant positive effects of PRP injections, as compared with placebo injections, in patients with muscle injuries, up to one year after injections (Reurink et al. 2014; Reurink et al. 2015). Customization of PRP preparation, as recently demonstrated by the use of TGF-β1 neutralizing antibodies, is a promising alternative to promote muscle regeneration while significantly reducing fibrosis (Li et al. 2016).

**Table 1:** The role of growth factors in skeletal muscle regeneration

Growth factors	Physiological effects, potential benefits	Shortcomings	Commentary
IGF-1	- Essential for muscle growth during development and regeneration. - Promote myoblast proliferation and differentiation in vitro (Huard et al. 2002) - Hypertrophic effect of IGF-1 (Barton-Davis et al. 1999) - Serial injections of IGF-1 improve muscle healing in vivo (Menetrey et al. 2000). - Existence of a muscle specific isoform of IGF-1 (mIGF-1) (Musaro et al. 1999; Musaro et al. 2004)	- Chemotactic for fibroblasts, increase collagen production, enhance fibrosis development	- IGF-1 play a central role in the enhancement of muscle regeneration- - Anti-inflammatory actions of IGF-1 (Mourkioti and Rosenthal 2005; Tidball and Welc 2015)
HGF	- Promote myoblast proliferation and inhibit myoblast differentiation (Anderson 2016; Yin et al. 2013) - Important role for satellite cell activation. Balance between the activation of satellite cells and their return to quiescence. (Chazaud 2010) - Recently, it was shown that a second set of HGF production is crucial for inflammation resolution after injury (Proto et al. 2015)	- Injection of HGF into injured muscle increased myoblast numbers but blocked the regeneration process (Miller et al. 2000)	- HGF is important during the early phase of muscle regeneration, activate satellite cells
VEGF	- Important signaling protein that favor angiogenesis. - Promote myoblast migration, proliferation and survival. (Arsic et al. 2004) - VEGF administration improves muscle regeneration. (Messina et al. 2007; Deasy et al. 2009)	- Non regulated VEGF expression promote aberrant angiogenesis and fibrosis in skeletal muscle (Karvinen et al. 2011)	- Importance of the proximity between satellite cells and the microvasculature during muscle regeneration, role of VEGF
FGF	- Large family of mitogen involved in cell growth and survival - FGF-6 has a muscle specific expression, stimulates satellite cell proliferation and promotes myogenic terminal differentiation (Floss et al. 1997) - FGF-2 promote satellite cell proliferation and inhibit myogenic differentiation (Menetrey et al. 2000; Kastner et al. 2000)	- Stimulate fibroblast proliferation,	- FGF signaling plays a key role in muscle repair, blocking FGF signaling delay muscle regeneration (Saera-Vila et al. 2016).
TGF-β1	- Key regulator of the balance between muscle fibrosis and muscle regeneration - Inhibits satellite cell proliferation and differentiation in vitro	- Excessive TGFβ1-induced deposition of ECM at the site of injury, fibrosis (Garg et al. 2015).	- Anti fibrotic therapy by blocking overexpression of TGF-β1 improve muscle regeneration. (Burks et al. 2011; Hwang et al. 2016)
PDGF-BB	- PDGF isoforms can regulate myoblast proliferation and differentiation in vitro (Yablonka-Reuveni et al. 1990) - PDGF-BB stimulates satellite cell proliferation and inhibit their differentiation (Charge and Rudnicki 2004)	- Potent mitogen for fibroblasts	- Release from injured vessels and platelets, PDGF stimulates early skeletal muscle regeneration

#### Stem cells

Transplantation of satellite cell-derived myoblasts has long been explored as a promising approach for treatment of skeletal muscle disorders. After an initial demonstration that normal myoblasts can restore dystrophin expression in *mdx* mice (Partridge et al. 1989), clinical trials, in which allogeneic normal human myoblasts were injected intramuscularly several times in dystrophic young boys muscles, have not been successful (Law et al. 1990; Mendell et al. 1995). Even recently, despite clear improvement in methodologies that enhance the success of myoblast transplantation in Duchenne patients (Skuk et al. 2007), outcomes of clinical trials are still disappointing. These experiments have raised concerns about the limited migratory and proliferative capacities of human myoblasts, as well as their limited life span in vivo. It led to the investigations of other muscle stem cells sources that could overcome these limitations and outperform the success of muscle cell transplantation. Among all these non-satellite myogenic stem cells, human mesoangioblasts, human myogenic-endothelial cells and human muscle–derived CD133+ have shown myogenic potentials in vitro and in vivo (Sampaolesi et al. 2006; Zheng et al. 2007; Meng et al. 2014). The use of such myogenic progenitors cells for improving muscle healing may become an interesting therapeutic alternative (Tedesco and Cossu 2012; Tedesco et al. 2010; Chen et al. 2012). A first phase I/IIa clinical trial has recently demonstrated that intra arterial injections of human mesoangioblasts are safe but display only very limited clinical efficacy in Duchenne patients (Cossu et al. 2015).

#### Scaffolds

Myogenic precursor cell survival and migration is greatly increased by using appropriate scaffold composition and growth factor delivery (Hill et al. 2006) (Boldrin et al. 2007). Controlling the microenvironment of injected myogenic cells using biological scaffolds enhance muscle regeneration (Borselli et al. 2011). Ideally, using an appropriate extracellular matrix (ECM) composition and stiffness, scaffolds should best replicate the in vivo milieu and mechanical microenvironment (Gilbert et al. 2010) (Engler et al. 2006). A combination of stem cells, biomaterial-based scaffolds and growth factors may provide a therapeutic option to improve regeneration of injured skeletal muscles (Jeon and Elisseeff 2016).

#### Anti-fibrotic therapy

TGF-β1 is expressed at high levels and plays an important role in the fibrotic cascade that occurs after the onset of muscle injury (Bernasconi et al. 1995; Li et al. 2004). Therefore, neutralization of TGF-β1 expression in injured skeletal muscle should inhibit the formation of scar tissue. Indeed, the use of anti-fibrotic agents (ie decorin, relaxin, antibody against TGF-β1…) that inactivate TGF-β1 signaling pathways reduces muscle fibrosis and, consequently, improve muscle healing, leading to a near complete recovery of lacerated muscle (Fukushima et al. 2001; Li et al. 2007). Losartan, an angiotensin II receptor antagonist, neutralize the effect of TGF-β1 and reduce fibrosis, making it the treatment of choice, since it already has FDA approval to be used clinically (Bedair et al. 2008; Park et al. 2012; Terada et al. 2013). Suramin, also approved by the FDA, blocks TGF-β1 pathway and reduces muscle fibrosis in experimental model (Chan et al. 2003; Taniguti et al. 2011).

#### Mechanical stimulation

Mechanical stimulation may offer a simple and effective approach to enhance skeletal muscle regeneration. Stretch activation, mechanical conditioning but also massage therapy or physical manipulation of injured skeletal muscles have shown multiple benefit effects on muscle biology and function in vitro and in vivo (Tatsumi et al. 2001);(Best et al. 2012) (Crane et al. 2012; Kumar et al. 2002; Gilbert et al. 2010; Powell et al. 2002). Recently, Cezar and colleagues demonstrates that mechanical forces are as important biological regulators as chemicals and genes, and underlines the immense potential of developing mechano-therapies to treat muscle damage (Cezar et al. 2016). A recent study also demonstrated that a treatment based on ultrasound-guided intra-tissue percutaneous electrolysis (EPI technique) enhances the treatment of muscle injuries (Abat et al. 2015). Altogether, these results suggest that mechanical stimulation should be considered as a possible therapy to improve muscle regeneration and repair.

## Conclusions

Skeletal muscle injuries are very frequently present in sports medicine and pose challenging problems in traumatology. Despite their clinical importance, the optimal rehabilitation strategies for treating these injuries are not well defined. After a trauma, skeletal muscles have the capacity to regenerate and repair in a complex and well-coordinated response. This process required the presence of diverse cell populations, up and down-regulation of various gene expressions and participation of multiples growth factors. Strategies based on the combination of stem cells, growth factors and biological scaffolds have already shown promising results in animal models. A better understanding of the cellular and molecular pathways as well as a better definition of the interactions (cell-cell and cell-matrix) that are essential for effective muscle regeneration, should contribute to the development of new therapies in humans. In this direction, a recent paper from Sadtler et al demonstrated that specific biological scaffold implanted in injured mice muscles trigger a pro-regenerative immune response that stimulate skeletal muscle repair (Sadtler et al. 2016).

## Abbreviation

CTGF, connective tissue growth factor; FGF, fibroblast growth factor; HGF, hepatocyte growth factor; IGF-I, insulin like growth factor-I; NMJ, neuromuscular junction; PDGF, platelet derived growth factor; PRP, platelet rich plasma; SC, satellite cells; TGF-β1, transforming growth factor β1; VEGF, vascular endothelial growth factor
